# Skeletal Muscle Quality Evaluation for Prognostic Stratification in the Emergency Department of Patients ≥65 Years with Major Trauma

**DOI:** 10.3390/jcm14217504

**Published:** 2025-10-23

**Authors:** Marcello Covino, Luigi Carbone, Martina Petrucci, Gabriele Pulcini, Marco Cintoni, Luigi Larosa, Andrea Piccioni, Gianluca Tullo, Davide Antonio Della Polla, Benedetta Simeoni, Mariano Alberto Pennisi, Antonio Gasbarrini, Maria Cristina Mele, Francesco Franceschi

**Affiliations:** 1 Emergency Medicine, Fondazione Policlinico Universitario A. Gemelli IRCCS, 00168 Rome, Italy; marcello.covino@policlinicogemelli.it (M.C.); andrea.piccioni@policlinicogemelli.it (A.P.); gianluca.tullo@policlinicogemelli.it (G.T.); benedetta.simeoni@policlinicogemelli.it (B.S.); francesco.franceschi@policlinicogemelli.it (F.F.); 2 Faculty of Medicine and Surgery, Università Cattolica del Sacro Cuore, 00168 Rome, Italy; marianoalberto.pennisi@policlinicogemelli.it (M.A.P.); antonio.gasbarrini@policlinicogemelli.it (A.G.); mariacristina.mele@policlinicogemelli.it (M.C.M.); 3 Department of Emergency Medicine, Ospedale Fatebenefratelli Isola Tiberina, Gemelli–Isola, 00166 Rome, Italy; luigi.carbone@fbf-isola.it; 4 Clinical Nutrition Unit, Fondazione Policlinico Universitario A. Gemelli IRCCS, 00168 Rome, Italy; gabriele.pulcini@policlinicogemelli.it (G.P.); marco.cintoni@policlinicogemelli.it (M.C.); 5 Department of Radiology, Fondazione Policlinico Universitario A. Gemelli IRCCS, 00168 Rome, Italy; luigi.larosa@policlinicogemelli.it; 6 Department of Anaesthesiology and Intensive Care Medicine, Fondazione Policlinico Universitario A. Gemelli IRCCS, 00168 Rome, Italy; 7 Department of Internal Medicine, Fondazione Policlinico Universitario A. Gemelli IRCCS, 00168 Rome, Italy

**Keywords:** emergency department, major trauma, trauma in elderly, Injury Severity Score, older adults, CT scan muscle quality assessment, skeletal muscle area, skeletal muscle density

## Abstract

**Background:** In patients aged 65 years and older who experience severe trauma, their underlying health status significantly influences overall mortality. This study aimed to determine whether computed tomography (CT) evaluation of skeletal muscle quality could serve as an effective risk stratification tool in the emergency department (ED) for this population. **Methods:** Retrospective observational study conducted between January 2018 and September 2021, including consecutive patients ≥65 years admitted to the ED for a major trauma (defined as having an Injury Severity Score > 15). Muscle quality analysis was made by specific software (Slice-O-Matic v5.0, Tomovision^®^, Montreal, QC, Canada) on a CT-scan slice at the level of the third lumbar vertebra (L3). **Results:** A total of 263 patients were included (72.2% males, median age 76 (71–82)), of whom 88 (33.5%) died during hospitalization. The deceased patients had a significantly lower skeletal muscle area density (SMAd) compared with survivors. The multivariate Cox regression analysis confirmed that SMAd <38 at the ED admission was an independent risk for death (adjusted HR 1.68 [1.1–2.7]). The analysis also revealed that, among the survivors after the first week of hospitalization, the patients with low SMAd had an increased risk of death (adjusted HR 3.12 [1.2–7.9]). **Conclusions:** Skeletal muscle density assessed by a CT scan at ED admission may represent a valuable prognostic marker for risk stratification patients ≥65 years with major trauma. In patients with SMAd <38 HU the in-hospital mortality risk could be particularly increased after the first week of hospitalization.

## 1. Introduction

The progressive aging of the global population poses substantial challenges to healthcare systems [1,2], as the number of older adults is increasing faster than that of any other age groups in nearly all countries [2]. Consequently, an increasing proportion of older individuals are presenting to the Emergency Department (ED) with traumatic injuries [3,4,5]. In the US in 2016, adults ≥65 years accounted for about 1/3 of all trauma patients, with a case fatality rate much higher than the younger population, and similar trends have been reported in the European Union [6,7].

Older adults with trauma often present a higher number of comorbidities and more complex clinical conditions compared to younger patients [8,9]. Regardless of other contributing factors, these patients are indeed more prone to experiencing heart, pulmonary, and renal complications [10,11]. Moreover, particularly when emergency surgery is required, overall mortality increases with age, doubling in patients aged ≥80 years old [12,13].

Nevertheless, chronological age and comorbidities do not always accurately reflect the overall health status of older patients. To better access this population, the concept of frailty has been introduced, defined as a state of increased vulnerability to stressors and characterized by a progressive declined physiological function and reduced strength, which together increase the risk of adverse outcomes [14,15]. However, a comprehensive evaluation of frailty requires a full geriatric assessment, which could be difficult to perform in the ED, particularly in cases of major trauma.

Sarcopenia, defined as the age-related loss of muscle mass and function [16,17] shares several clinical features with frailty [18] and may serve as a marker of increased frailty. Both sarcopenia and frailty are closely associated with poor nutritional status [19], and have been linked to worse clinical outcomes in elderly patients [20,21,22]. Various methods can be used to evaluate sarcopenia, and radiology plays a prominent role, even though there is a lack of consensus on their standardization [23,24]. Nevertheless, computed tomography (CT) offers an objective and reproducible means of assessing both sarcopenia and overall frailty [25].

This study aims to investigate the association between skeletal muscle quality, as assessed by CT imaging, and mortality in older patients admitted to the ICU following major trauma. Specifically, the study seeks to determine whether CT-derived measures of muscle density and mass can serve as prognostic indicators of short- and long-term outcomes in this population.

A better understanding of the relationship between muscle quality and mortality could help identify high risk geriatric trauma patients early in their clinical course. Incorporating radiological indicators of sarcopenia into trauma assessment protocols could facilitate more accurate prognostic stratification, guide tailored nutritional and rehabilitation intervention, and improve patient outcomes.

## 2. Materials and Methods

This is a monocentric retrospective observational cohort study conducted in the ED of a teaching hospital in central Italy, with a catchment area of about 1.8 M inhabitants, and about 80k patients admitted per year. The institution held a trauma center treating about 2k major traumas per year.

This retrospective observational study aimed at evaluating the association between radiological parameters and in-hospital mortality.

The study enrolled all consecutive patients ≥65 years who were admitted to our ED for major trauma between January 2018 and September 2021.

The definition of major trauma is primarily based on anatomical and physiological criteria, as well as resource utilization. In international settings and trauma systems, major trauma is traditionally identified as an injury resulting in an Injury Severity Score (ISS) ≥16.

This score reflects the severity of injuries across multiple body regions and is associated with an increased risk of mortality and the need for intensive care.

Given the observational nature of the study and its retrospective design, no a priori sample size calculation was performed. Instead, we included all eligible consecutive patients aged ≥65 years who presented with major trauma (ISS ≥ 16) and met the inclusion criteria during the predefined study period (January 2018 to September 2021). This approach was chosen to ensure the representativeness of the sample and to reduce selection bias.

The final sample size reflects the real-world incidence of major trauma among older adults presenting to our Emergency Department, which is a high-volume trauma center with approximately 2000 major trauma cases annually. Stratification into outcome-based groups (e.g., survivors vs. non-survivors; early vs. late mortality) was performed post hoc, based on clinical endpoints.

For each patient, hospital-based, electronic health records were used to collect all the demographic and clinical data. Patients with minor injuries were excluded from the study. Additional exclusion criteria included the absence of an abdominal CT scan at ED admission and the presence of severe intramuscular hemorrhage at the level of the third lumbar vertebra, which could impair accurate muscle assessment on CT.

Quantification Method: Quantification of body composition parameters was performed on a single axial CT slice at the level of the third lumbar vertebra (L3). Using the “Slice-O-Matic v5.0” (Tomovision^®^, Montreal, QC, Canada), the cross-sectional areas of skeletal muscle (SMA), subcutaneous adipose tissue (SAT), and visceral adipose tissue (VAT) were segmented according to predefined Hounsfield Unit (HU) thresholds: SMA −29 to +150 HU, SAT −190 to −30 HU, and VAT −150 to −50 HU.

For each region of interest (ROI), the mean HU value was automatically calculated by the software to obtain tissue density values (SMAd, SATd, and VATd). All measurements were independently performed by two trained investigators with over five years of imaging experience, blinded to clinical outcomes. The average of the two measurements was used for statistical analysis to minimize interobserver variability and reduce bias.

### 2.1. Study Variables

Upon presentation to the ED, for each patient, the following pieces of information were collected for each patient:

- Demographic data, including age and sex.
- Physiological parameters at ED admission including Glasgow Coma Scale, respiratory rate, systolic blood pressure.
- Acute Injury Scale (AIS) scores and the Injury Severity Score (ISS). The scores were blindly calculated for each patient by three authors (MP, LF, GT) based on the clinical records and the radiological findings.
- Information about clinical history and comorbidities was assessed with the Charlson Comorbidity Index (CCI), a validated score used to predict the risk of death one year after hospitalization in patients with a high comorbidities burden.
- The average length of stay (LOS) was calculated from the time of the ED admission to the time of discharge or death.
- Laboratory tests, including hemoglobin, white blood cells, platelet count, fibrinogen, prothrombin time, partial thromboplastin time, glucose, creatinine, urea, nitrogen, and blood gas analysis results (pH, lactates, bicarbonates).
- Assessment of muscle quality. Body composition analysis was performed on a single axial CT-scan slice (DICOM image format) at the level of the third lumbar vertebra (L3), using specific software (Slice-O-Matic v5.0, Tomovision^®^, Montreal, QC, Canada). Image analysis was performed by two investigators with over five years imaging experience and blinded to outcomes, to minimize the introduction of bias. The cross-sectional area of skeletal muscle (SMA), subcutaneous adipose tissue (SAT), and visceral adipose tissue (VAT) were analyzed based on pre-established thresholds of Hounsfield Units (HU): SMA −29 to 150, SAT −190 to −30, and VAT −150 to −50. Skeletal muscle area density (SMAd) was calculated by finding the mean of the HU of SMA. Similarly, the mean HU density was calculated for VAT (VATd) and SAT (SATd) [21]. Supplementary Figure S1 shows a sample of the CT images used for the calculations.

### 2.2. Study Endpoints

The primary study endpoint of this study was all-cause in-hospital death.

Secondary endpoints included early mortality (defined as mortality within 7 days since admission) and the late mortality (defined as mortality that occurred >7 days since admission).

### 2.3. Statistical Analysis

This was a retrospective observational study aimed at evaluating the association between radiological parameters and in-hospital mortality.

Continuous variables were reported as a median [interquartile range, IQR] and compared using univariate analysis by the Mann–Whitney U test. Categorical variables were expressed as absolute numbers (percentage) and were compared using the Chi-square test (with Fisher’s test if appropriate). Receiver operating characteristic (ROC) curve analysis was used to estimate the performance of the evaluated radiological parameters in predicting in-hospital death. The Youden index was used to estimate the optimal cut-off threshold associated with the defined outcomes. The areas under the ROC curve were compared by the DeLong method. Significant variables in the univariate analysis were entered into a multivariate Cox regression model to identify the independent predictors of in-hospital death. The single items included in combined variables (i.e., APACHE II score and CCI) were not included in multivariate models to avoid overfitting and factor overestimation. Furthermore, to improve the parameter estimation and the model fitting, the continuous variables were dichotomized according to the cut-off values identified by ROC analysis. Early and late mortality were evaluated in separate multivariate Cox regression models, by considering only the events that occurred at <7 days and ≥7 days, respectively, and considering “censored” cases as the remaining deaths. A *p*-value of 0.05 was regarded as significant in all the analyses.

A Cox proportional hazards model was used because it allows the assessment of the association between multiple variables and the risk of in-hospital death over time, taking into account both the timing of the event and censored cases.

Data were analyzed using IBM SPSS statistics for Windows, Version 25 (IBM Corp., Armonk, NY, USA) and MedCalc Statistical Software version 19.2.1 (MedCalc, Ostend, Belgium).

### 2.4. Ethical Approval

The study was conducted according to the principles expressed in the Declaration of Helsinki and its later amendments. All patients gave their informed consent to participate in the study. The research protocol was approved by the Institutional Review Board of Fondazione Policlinico Universitario “A Gemelli” IRCCS—Rome (#0025817/22; Study ID: #5121).

## 3. Results

### 3.1. Study Cohort and Baseline Characteristic

A total of 263 patients (179 males, 72.2%) were enrolled in the study. The median age was 76 years (71–82). The enrolled patients had a median ISS 26 (20–33); the AIS was highest for head and neck injuries (three, ranging from two to five). As expected, the enrolled patients had several comorbidities, and the median CCI was four (3–5). Overall, 175 patients survived (66.5%), with a median length of hospital stay (LOS) of 15.5 days (6.2–29.4) (Table 1).

The 88 patients deceased during hospitalization where significantly older than survivors, although gender distribution and overall comorbidity burden, as measured by CCI, were similar between the groups (Table 1). As expected, deceased patients had a higher ISS compared to survivors (19 (25–33) vs. 25 (17–33), *p* < 0.001), with the most pronounced differences observed in head and neck injuries. Similarly, the APACHE II score was significantly higher in non-survivors compared to survivors (27 (24–30) vs. 17 (14–21). *p* < 0.001) (Table 1).

Among the laboratory parameters assessed at the ED admission, deceased patients had lower Hb values and higher derangement of coagulation (Table 1), reflecting the possible relationship between acute bleeding and worse outcomes.

### 3.2. Muscular Quality Assessment

Comparing the muscular and adipose tissue at the level of the third lumbar between the deceased and survivors, the two groups had similar SM, VAT, and SAT areas (Table 1). However, the mean tissue density as expressed by Hounsfield units (HU), was significantly different between the groups. The deceased had lower SMA density (37.9 (32.2/45.7) vs. 41.9 (35.9/48.1) vs. *p* = 0.009), and higher VAT density (−82.5 (−86.1/−75.6) vs. −83.9 (−87.9/−79.5), *p* = 0.047), and SAT density (−82.6 (−87.8/−77.8) vs. −85.7 (−89.8/−80.0), *p* = 0.029) (Table 1).

Evaluating these parameters by ROC analysis, SMAd showed the best association with in-hospital death with an AUROC of 0.599, followed by SATd and VATd (AUROC 0.582 and 0.575, respectively) (Table 2). There were no significant differences among the AUROC (SMAd vs. SATd z statistic 0.326, *p* = 0.744; SMAd vs. VATd z statistic 0.458, *p* = 0.657; VATd vs. SATd z statistic 0.220, *p* = 0.825). According to the Youden index J, the best discriminating values for the prediction of in-hospital death were <38 HU for SMAd, >−77 HU for VATd, and >−83 HU for SATd. However, these thresholds had limited sensitivity and specificity (Table 2).

### 3.3. Multivariate Analysis for Survival

**Figure 1:**
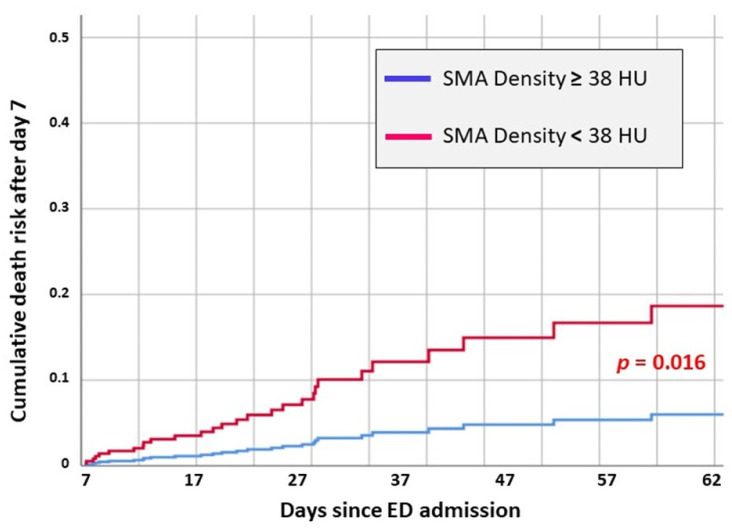
Survival curves comparison of major trauma patients ≥65 years based on different skeletal muscle area density (SMAd) measured by CT scans at the level of the third lumbar vertebrae. Muscular density is expressed in Hounsfield Units (HU). The adjusted hazard ratio for patients with SMAd <38 HU was 1.68 [1.06–2.67], *p* = 0.028.

The multivariate Cox regression model revealed that SMAd <38 HU was an independent risk factor for in-hospital death in our cohort (HR 1.68 [1.02–1.05]) (Figure 1).

Together with SMAd, independent risk factors for poor outcomes were as follows: ISS > 24 (HR 2.8, an increased aPTT at ED admission (HR 1.04 [1.02–1.05]), a higher injury severity according to ISS (HR 1.02 [1.01–1.04]), and older age (HR 1.04 [1.01–1.08]) (Table 3, Figure 1).

### 3.4. Early and Late Mortality Analysis

**Figure 2:**
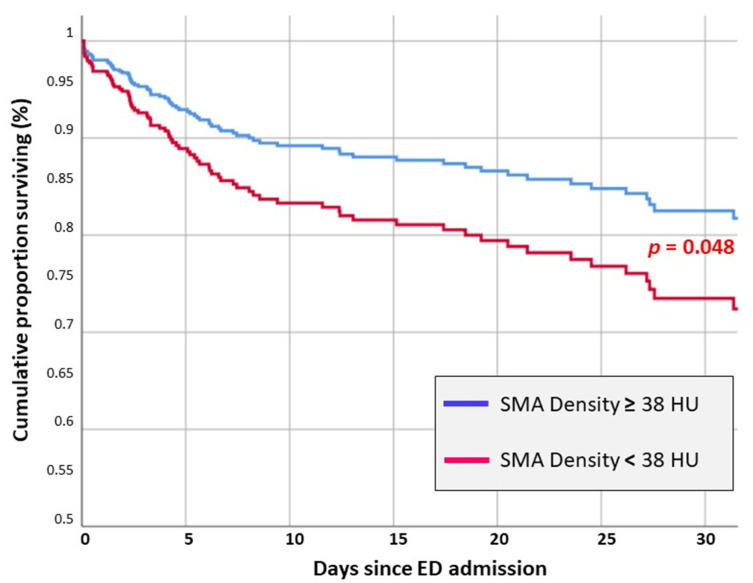
Overall survival.

When considering only the patients deceased within the first week since admission, the Cox regression analysis confirmed APACHE II score > 22, ISS > 24, and aPTT > 31.6 as independent risk factors for in-hospital death. In these patients, muscle quality parameters were not significantly associated with death (Table 4, Figure 2).

Interestingly, when considering only the deaths in patients surviving the first week of hospitalization, only APACHE II >22 and SMAd <38 were associated with in-hospital death (Table 4). In particular, the analysis revealed that patients with SMAd <38 had about a three-fold increased risk of death during the later phase of the hospitalization (HR 3.12 [1.23–7.88]), independent of other clinical factors.

## 4. Discussion

The main finding of the present study is that in patients aged ≥ 65 years with major trauma, reduced skeletal muscle density was independently associated with in-hospital mortality, regardless of other clinical characteristics.

Although the role of muscle mass in trauma patients is well established, most studies have focused on the recovery phase, linking higher muscle mass to better outcomes [26]. However, this is mainly due to the known loss of muscle mass during prolonged hospitalization and does not specifically address muscle quality [27].

In a 2021 study by Xi et al., it was reported that in patients who experienced abdominal trauma, poorer skeletal muscle quality was linked to increased length of hospital stay and increased number of complications [28]. Although definitive conclusions cannot be drawn, pre-existing nutritional status is likely to play a contributory role. The importance of nutrition in preserving skeletal muscle is well known [29] and nutritional interventions are now a mainstay in the treatment of elderly patients who are affected by sarcopenia. It could be tempting to suggest that poor skeletal muscle quality is simply a proxy for malnutrition, which is the real determinant for worse clinical outcomes, but it is likely that other mechanisms could also play a role [30,31].

Muscle quality and mass are influenced by systemic inflammation, which impairs regeneration and promotes fat accumulation, further exacerbating inflammation [31,32,33]. Inflammation also promotes the increase in adipose tissue, which could be in itself a trigger for further inflammation [33]. Both increased adiposity and inflammation have been linked to complications and worse outcomes in trauma patients [30].

Also, it has been speculated that muscle mass could be associated with social and economic factors, which could in turn play a role in the worse outcomes experienced by this group of patients [34,35]. However, our study does not include data to explore or confirm this hypothesis.

An additional interesting finding was the different adipose tissue distribution (both visceral and subcutaneous) in deceased patients (Table 1).

Although adipose tissue in the elderly population has been associated with increased inflammation [33], it is important to note that reduced adipose tissue may also result from malnutrition [36]. On the other hand, some authors have reported that contrary to common sense, an increased adipose tissue could be linked to malnutrition and sarcopenia [37]. However, the data derived from our cohort does not give any final clues on this point. In our cohort, while the SATd and VATd values were significantly different between survivors and controls (Table 1 and Table 2), the characteristics of the adipose tissue did not prove to be independent risk factors for poor outcomes in the multivariate analysis (Table 3).

Interestingly, our analysis showed that short-term mortality (<7 days) was mostly primarily correlated with trauma severity, advanced age, and coagulation abnormalities (Table 4). Late mortality was higher in the group with poor muscle quality, and the difference was higher as the length of hospital stay increases (Figure 1). Interestingly, the rate of infectious complications such as pneumonia and sepsis was not significantly different among those who survived and those who did not, suggesting that low muscle quality in itself may have played a key role in increasing mortality. As is well known, sarcopenia increases the risk of experiencing trauma in geriatric patients, which is associated with a significant burden in terms of mortality and morbidity in modern societies [38].

In our cohort, the skeletal muscle quality, and specifically the overall muscle density, was significantly associated with late mortality. While it is somewhat intuitive that patients with higher muscle mass may recover better from trauma than sarcopenic patients, our data revealed that good muscle quality may be an even more important predictor of outcomes than traditionally recognized factors such as comorbidities and advanced age.

CT-based skeletal muscle density measurement, easily obtainable from routine trauma imaging, may serve as a novel prognostic biomarker to refine outcome prediction, optimize resource allocation, and personalize the management of trauma patients, particularly in the geriatric population.

The study also confirmed that the APACHE II score is a reliable tool for stratifying mortality risk in trauma patients ≥ 65 years [39]. As expected, both the type and severity of trauma were strongly associated with clinical outcomes. In particular, higher ISS values where significantly correlated with increased mortality, indicating that overall trauma burden has a direct impact on prognosis. Similarly, elevated AIS scores for the head and neck were significantly associated with worse mortality rates [40,41]. Lastly, the factors associated with acute bleeding such as lower Hb and an increased aPTT, showed a high correlation with in-hospital mortality in our cohort, particularly for the early mortality cases (Table 3 and Table 4). Indeed, although rapid control of bleeding and coagulation are currently the mainstay of trauma resuscitation, the overall mortality among bleeding trauma patients is still high [42]. Moreover, the mortality of these patients could not be limited to the very short term due to exsanguination but can also occur later in the clinical course [42,43,44,45]. On the other hand, it is worth noting that in older adults the use of anticoagulants is more common than in the general population and is associated with increased bleeding risk, overall increased mortality, and the need for a post-hospital care facility [46].

The burden of comorbidities is usually considered one of the main determinants of worse outcomes in trauma patients, particularly for older adults. As reported in a study by Gioffrè-Florio et al. [38], comorbidities such as osteoporosis are linked to worse outcomes in patients who experience a fall. At the same time, other authors have even designed specific comorbidity indexes for trauma patients [47]. In our population, we observed that chronic kidney disease (CKD) was the only comorbidity associated with an increase in mortality, but the overall number of comorbidities, evaluated through the CCI, was not significantly higher. This latter finding, which is not consistent with the literature, could be due to the specific older population, and to the different methods used to assess comorbidity in different studies [48]. Interestingly, in our sample, patients who experienced higher mortality showed lower levels of hemoglobin. Anemia has been known to be associated with higher mortality in elderly trauma patients and has been linked to a higher prevalence of CKD, which could be consistent with our population [49]. At the same time, CKD is linked to sarcopenia, and its development in patients with CKD is multifactorial and it may occur independently of weight loss or cachexia [50].

## 5. Limitations

Our study presented some limitations, particularly due to its retrospective design, which does not allow us to draw any definite conclusions on the causal link between skeletal muscle quality and prognosis. At the same time, although we found an association, the mechanisms underlying it are not clear and were not investigated in the present study. Finally, while the overall severity of trauma and multiple clinical parameters were taken into consideration, still several potential confounders could not be addressed in the present analysis. In particular, key variables related to the patient’s inflammatory status and nutritional condition—which may significantly influence both muscle quality and clinical outcomes—were not available in the dataset and could not be assessed.

## 6. Conclusions

Our study demonstrated that in patients ≥65 years admitted to the ED for major trauma, CT-based quality assessment at admission may serve as an effective tool for prognostic risk stratification.

Moreover, prognosis appears to be influenced not merely by muscle mass, but primarily by muscle quality, as assessed by average muscle density on a CT scan. This finding is particularly relevant because muscle quality is closely associated with nutritional status, underscoring the potential role of nutrition in the management of elderly trauma patients.

Future studies should collect nutritional, inflammatory, and rehabilitation data to explore the underlying mechanisms linking muscle quality, systemic response, and clinical outcomes.

Overall, reduced skeletal muscle quality (SMAd < 38 HU) was demonstrated to be an independent risk factor for in-hospital mortality in older adults, particularly in the late phase of the clinical course.

## Figures and Tables

**Table 1:** Clinical characteristics of the patients in the study cohort and comparison between deceased vs. survivors.

	All Patients n 263	Survived n 175	Deceased n 88	*p* Value
Age	76 (71–82)	75 (69–81)	78 (74–85)	**<0.001**
Sex (male)	179 (72.2%)	132 (75.4%)	47 (64.4%)	0.088
*Injury severity*				
*ISS*	26 (20–33)	25 (17–33)	29 (25–33)	**<0.001**
*AIS Head Neck*	*3 (2–5)*	*3 (0–4)*	*5 (4–5)*	<0.001
*AIS Face*	0 (0–2)	0 (0–2)	0 (0–1.5)	0.724
*AIS Chest*	2 (0–4)	2 (0–4)	0 (0–3)	0.066
*AIS Abdomen*	0 (0–0)	0 (0–2)	0 (0–0)	0.124
*AIS Pelvic-Extremity*	0 (0–2)	1 (0–3)	0 (0–2)	0.028
*AIS External*	0 (0–1)	1 (0–1)	0 (0–1)	0.027
APACHE II	20 (15–26)	17 (14–21)	27 (24–30)	**<0.001**
*Laboratory values at ED admission*				
*Hb* (mg/dL)	12.8 (11.2–14)	13.1 (11.4–14.2)	11.9 (10.2–13.5)	**<0.001**
*WBC* (×10^9^)	13.2 (9.19–16.84)	13.3 (9.48–16.47)	12.6 (9–19.5)	0.638
*PLT*	205 (156–257)	208 (169–253)	183 (148–268)	0.319
*Fibrinogen*	287 (250–335)	285 (252–327)	290 (244–365)	0.421
*aPTT*	28.6 (25.5–34.4)	27.2 (24.9–31.7)	33.6 (28.5–39.5)	**<0.001**
*Glucose*	158 (133–207)	152 (131–200)	170 (135–209)	0.081
*Creatinine* (mg/dL)	1.01 (0.8–1.26)	1 (0.8–1.23)	1.05 (0.8–1.36)	0.437
*BUN* (mg/dL)	20 (17–24)	20 (17–24)	20.5 (18–28.8)	0.574
*Lactate* (mmol/L)	2.5 (1.8–3.5)	2.5 (1.8–3.4)	2.7 (1.7–7.8)	0.725
*Muscular parameters at CT scan evaluation*				
*SMA*	151.2 (122.5/170.5)	153.8 (127.7/169.8)	140.5 (112.3/173.9)	0.204
*VAT Area*	144.3 (77.2/218.9)	158.9 (87.9/222.3)	128.3 (77.9/202.1)	0.112
*SAT Area*	150.9 (110.2/207.5)	154.4 (113.3/211.45)	142 (98.67/191.1)	0.119
*SMA Density*	41.6 (34.72/47.8)	41.9 (35.9/48.1)	37.9 (32.2/45.7)	**0.009**
*VAT Density*	−83.4 (−87.2/−78.0)	−83.9 (−87.9/−79.5)	−82.5 (−86.1/−75.6)	**0.047**
*SAT Density*	−85.0 (−89.0/−78.9)	−85.7 (−89.8/−80.0)	−82.6 (−87.8/−77.8)	**0.029**
*Comorbidities*				
*CCI*	4 (3–5)	4 (3–5)	4 (3–5)	0.053
*History of CAD*	19 (7.7%)	14 (8%)	5 (6.8%)	1.000
*Congestive Heart Failure*	5 (2%)	2 (1.1%)	3 (4.1%)	0.154
*Peripheral Vascular Disease*	26 (10.5%)	17 (9.7%)	9 (12.3%)	0.649
*Cerebrovascular Disease*	13 (5.2%)	8 (4.6%)	5 (6.8%)	0.534
*Dementia*	8 (3.2%)	5 (2.9%)	3 (4.1%)	0.696
*COPD*	30 (12.1%)	25 (14.3%)	5 (6.8%)	0.134
*Diabetes*	3 (13.7%)	24 (13.7%)	10 (13.7%)	1.000
*Chronic Kidney Disease*	20 (8.1%)	10 (5.7%)	10 (13.7%)	0.043
*Malignancy*	17 (6.9%)	13 (7.4%)	4 (5.5%)	0.784

Abbreviations: Hb—Hemoglobin; WBC—White blood cells; PLT—Platelets; aPTT—activated Partial Thromboplastin Time; APACHE—Acute Physiology and Chronic Health Evaluation; BUN—Blood Urea Nitrogen; AIS—Acute Injury Scale; ISS—Injury Severity Score; CCI—Charlson Comorbidity Index; COPD—Chronic Obstructive Pulmonary Disease; SMA—Skeletal muscle area; VAT—Visceral adipose tissue; SAT—Subcutaneous adipose tissue.

**Table 2:** Receiver operating characteristic (ROC) analysis for the association between continuous variables and in-hospital death. The Youden index J was used to find the optimal cut-off value to dichotomize the variable.

	ROC Curve Area	p Value	Youden Index Cut-Off Value	Sensitivity [95% CI]	Specificity [95% CI]
Age	0.638 [0.577–0.696]	**<0.001**	>75	69.3 [58.6–78.7]	52.0 [44.3–59.6]
ISS	0.649 [0.588–0.707]	**<0.001**	<24	82.9 [73.4–90.1]	42.9 [35.4–50.5]
APACHE II	0.939 [0.902–0.964]	**<0.001**	>22	82.9 [73.4–90.1]	86.9 [80.9–91.5]
aPTT	0.727 [0.669–0.780]	**<0.001**	>31.6	64.7 [53.9–74.7]	74.8 [67.8–81.1]
*Muscular CT scan parameter*					
SMA Density	0.599 [0.537–0.658]	**0.038**	<38	52.3 [41.4–63.0]	73.1 [65.9–79.6]
VAT Density	0.575 [0.513–0.635]	**0.047**	>−77	31.8 [22.3–42.6]	82.8 [76.4–88.1]
SAT Density	0.582 [0.520–0.643]	**0.026**	>−83	51.4 [40.2–61.9]	63.4 [55.8–70.6]

Abbreviations: ISS—Injury Severity Score; aPTT—activated Partial Thromboplastin Time; APACHE—Acute Physiology and Chronic Health Evaluation; SMA—Skeletal muscle area; VAT—Visceral adipose tissue; SAT—Subcutaneous adipose tissue.

**Table 3:** Multivariate Cox regression analysis of the variables associated with all-cause in-hospital death. To improve model fitting and parameter estimation, the continuous variables were dichotomized by a cut-off chosen by ROC analysis with Youden index J.

Variable	Beta	Wald	Odds Ratio [95% CI]	*p*
Age > 75 years	0.327	1.330	1.39 [0.79–2.42]	0.249
ISS > 24	1.054	12.905	2.87 [1.61–5.10]	**<0.001**
APACHE II > 22	2.048	45.426	7.75 [4.27–14.07]	**<0.001**
aPTT > 31.6	0.579	5.251	1.78 [1.09–2.93]	**0.022**
SMA Density < 38 HU	0.519	4.836	1.68 [1.06–2.67]	**0.028**
SAT Density > −83 HU	0.149	0.311	1.16 [0.68–1.96]	0.577
VAT Density > −77 HU	0.197	0.466	1.22 [0.69–2.14]	0.495

Abbreviations: aPTT—activated Partial Thromboplastin Time; ISS—Injury Severity Score; HU—Hounsfield units; SMA—Skeletal muscle area; VAT—Visceral adipose tissue; SAT—Subcutaneous adipose tissue.

**Table 4:** Multivariate Cox regression analysis of the variables associated with the risk of in-hospital death (all-cause) within (model 1) and after (model 2) seven days since admission.

**Model 1**—Factors affecting mortality risk within 7 days since admission				
**Variable**	**Beta**	**Wald**	**Hazard Ratio [95% CI]**	***p***
Age > 75	0.632	3.837	1.88 [1.00–3.54]	0.050
ISS > 24	1.320	10.186	3.74 [1.66–8.41]	**0.001**
APACHE II > 22	1.528	18.736	4.61 [2.31–9.20]	**<0.001**
aPTT > 31.6	0.684	5.019	1.98 [1.09–3.61]	**0.025**
SMA Density < 38 HU	0.239	0.724	1.27 [0.73–2.20]	0.395
SAT Density > −83 HU	−0.047	0.019	0.89 [0.48–1.88]	0.891
VAT Density > −77 HU	−0.243	0.408	0.78 [0.37–1.65]	0.523
**Model 2**—Factors affecting mortality risk starting from 7 days since admission. The cases deceased within days were considered as “censored” in this regression model.				
**Variable**	**Beta**	**Wald**	**Hazard Ratio [95% CI]**	***p***
Age > 75	0.167	0.162	1.18 [0.52–2.66]	0.688
ISS > 24	0.669	2.370	1.95 [0.83–4.57]	0.124
APACHE II > 22	3.096	22.637	22.11 [6.18–79.15]	**<0.001**
aPTT > 31.6	0.064	0.016	1.06 [0.39–2.90]	0.900
SMA Density < 38 HU	1.137	5.771	3.12 [1.23–7.88]	**0.016**
SAT Density > −83 HU	−0.350	0.386	0.71 [0.23–2.12]	0.534
VAT Density > −77 HU	−0.019	0.001	0.98 [0.29–3.34]	0.976

Abbreviations: aPTT—activated Partial Thromboplastin Time; ISS—Injury Severity Score; HU—Hounsfield units; SMA—Skeletal muscle area; VAT—Visceral adipose tissue; SAT—Subcutaneous adipose tissue.

## Data Availability

Data will be available upon reasonable request from the corresponding author.

## Supplementary Materials

The following supporting information can be downloaded at: https://www.mdpi.com/article/10.3390/jcm14217504/s1. Figure S1: Abdominal CT scan at the level of the third lumbar in 72-year-old patients with major trauma, sample (A). Figure (B) shows the distribution of skeletal muscle area, visceral adipose tissue, and subcutaneous adipose tissue.

## Abbreviations

The following abbreviations are used in this manuscript:

ED	Emergency Department
CT	Computed Tomography
ISS	Injury Severity Score
AIS	Acute Injury Scale
CCI	Charlson Comorbidity Index
LOS	Length of Stay
SMA	Skeletal Muscle Area
SAT	Subcutaneous Adipose Tissue
VAT	Visceral Adipose Tissue
HU	Hounsfield Units
SMAd	Skeletal Muscle Area Density
VATd	Visceral Muscle Area Density
IQR	Interquartile Range
ROC	Receiver Operating Characteristic
CKD	Chronic Kidney Disease

## References

[1] Bengtsson T., Scott K. (2011). Population aging and the future of the welfare state: The example of Sweden. Popul. Dev. Rev..

[2] Kanasi E., Ayilavarapu S., Jones J. (2016). The aging population: Demographics and the biology of aging. Periodontol. 2000.

[3] Shenvi C.L., Platts-Mills T.F. (2019). Managing the Elderly Emergency Department Patient. Ann. Emerg. Med..

[4] Bonne S., Schuerer D.J. (2013). Trauma in the older adult: Epidemiology and evolving geriatric trauma principles. Clin. Geriatr. Med..

[5] Joseph A. (2015). Trauma in the elderly: Burden or opportunity?. Injury.

[6] European Union Eurostat-Accidents and Injuries Statistics. https://ec.europa.eu/eurostat/statistics-explained/index.php?title=Accidents_and_injuries_statistics#Deaths_from_accidents.2C_injuries_and_assault.

[7] American College of Surgeon-National Trauma Database Registry. https://www.facs.org/quality-programs/trauma/quality/national-trauma-data-bank/reports-and-publications/.

[8] Fogel J.F., Hyman R.B., Rock B., Wolf-Klein G. (2000). Predictors of hospital length of stay and nursing home placement in an elderly medical population. J. Am. Med. Dir. Assoc..

[9] Stonko D.P., Etchill E.W., Giuliano K.A., DiBrito S.R., Eisenson D., Heinrichs T., Morrison J.J., Haut E.R., Kent A.J. (2021). Failure to Rescue in Geriatric Trauma: The Impact of Any Complication Increases with Age and Injury Severity in Elderly Trauma Patients. Am. Surg..

[10] Loftus T.J., Brakenridge S.C., Murphy T.W., Nguyen L.L., Moore F.A., Efron P.A., Mohr A.M. (2018). Anemia and blood transfusion in elderly trauma patients. J. Surg. Res..

[11] McMahon D.J., Schwab C.W., Kauder D. (1996). Comorbidity and the elderly trauma patient. World J. Surg..

[12] Aucoin S., McIsaac D.I. (2019). Emergency General Surgery in Older Adults: A Review. Anesthesiol. Clin..

[13] Fu C.Y., Bajani F., Bokhari M., Starr F., Messer T., Kaminsky M., Dennis A., Schlanser V., Mis J., Poulakidas S. (2022). Age itself or age-associated comorbidities? A nationwide analysis of outcomes of geriatric trauma. Eur. J. Trauma Emerg. Surg. Off. Publ. Eur. Trauma Soc..

[14] Fried L.P., Tangen C.M., Walston J., Newman A.B., Hirsch C., Gottdiener J., Seeman T., Tracy R., Kop W.J., Burke G. (2001). Frailty in older adults: Evidence for a phenotype. J. Gerontol. Ser. A Biol. Sci. Med. Sci..

[15] Alqarni A.G., Gladman J.R.F., Obasi A.A., Ollivere B. (2023). Does frailty status predict outcome in major trauma in older people? A systematic review and meta-analysis. Age Ageing.

[16] Marzetti E., Calvani R., Tosato M., Cesari M., Di Bari M., Cherubini A., Collamati A., D’Angelo E., Pahor M., Bernabei R. (2017). Sarcopenia: An overview. Aging Clin. Exp. Res..

[17] Tagliafico A.S., Bignotti B., Torri L., Rossi F. (2022). Sarcopenia: How to measure, when and why. La Radiol. Medica.

[18] Picca A., Coelho-Junior H.J., Calvani R., Marzetti E., Vetrano D.L. (2022). Biomarkers shared by frailty and sarcopenia in older adults: A systematic review and meta-analysis. Ageing Res. Rev..

[19] Meza-Valderrama D., Marco E., Dávalos-Yerovi V., Muns M.D., Tejero-Sánchez M., Duarte E., Sánchez-Rodríguez D. (2021). Sarcopenia, Malnutrition, and Cachexia: Adapting Definitions and Terminology of Nutritional Disorders in Older People with Cancer. Nutrients.

[20] Xia L., Zhao R., Wan Q., Wu Y., Zhou Y., Wang Y., Cui Y., Shen X., Wu X. (2020). Sarcopenia and adverse health-related outcomes: An umbrella review of meta-analyses of observational studies. Cancer Med..

[21] Covino M., Salini S., Russo A., De Matteis G., Simeoni B., Maccauro G., Sganga G., Landi F., Gasbarrini A., Franceschi F. (2022). Frailty Assessment in the Emergency Department for Patients ≥80 Years Undergoing Urgent Major Surgical Procedures. J. Am. Med. Dir. Assoc..

[22] Covino M., Russo A., Salini S., De Matteis G., Simeoni B., Della Polla D., Sandroni C., Landi F., Gasbarrini A., Franceschi F. (2021). Frailty Assessment in the Emergency Department for Risk Stratification of COVID-19 Patients Aged ≥80 Years. J. Am. Med. Dir. Assoc..

[23] Chianca V., Albano D., Messina C., Gitto S., Ruffo G., Guarino S., Del Grande F., Sconfienza L.M. (2022). Sarcopenia: Imaging assessment and clinical application. Abdom. Radiol..

[24] Ardito F., Coppola A., Rinninella E., Razionale F., Pulcini G., Carano D., Cintoni M., Mele M.C., Barbaro B., Giuliante F. (2022). Preoperative Assessment of Skeletal Muscle Mass and Muscle Quality Using Computed Tomography: Incidence of Sarcopenia in Patients with Intrahepatic Cholangiocarcinoma Selected for Liver Resection. J. Clin. Med..

[25] Bardoscia L., Besutti G., Pellegrini M., Pagano M., Bonelli C., Bonelli E., Braglia L., Cozzi S., Roncali M., Iotti C. (2022). Impact of low skeletal muscle mass and quality on clinical outcomes in patients with head and neck cancer undergoing (chemo)radiation. Front. Nutr..

[26] Sueyoshi Y., Ogawa T., Koike M., Hamazato M., Hokama R., Tokashiki S., Nakayama Y. (2022). Improved activities of daily living in elderly patients with increased skeletal muscle mass during vertebral compression fracture rehabilitation. Eur. Geriatr. Med..

[27] Fazzini B., Märkl T., Costas C., Blobner M., Schaller S.J., Prowle J., Puthucheary Z., Wackerhage H. (2023). The rate and assessment of muscle wasting during critical illness: A systematic review and meta-analysis. Crit. Care.

[28] Xi F., Tan S., Gao T., Ding W., Sun J., Wei C., Li W., Yu W. (2021). Low skeletal muscle mass predicts poor clinical outcomes in patients with abdominal trauma. Nutrition.

[29] Gomes M.J., Martinez P.F., Pagan L.U., Damatto R.L., Cezar M.D.M., Lima A.R.R., Okoshi K., Okoshi M.P. (2017). Skeletal muscle aging: Influence of oxidative stress and physical exercise. Oncotarget.

[30] Chazaud B. (2020). Inflammation and Skeletal Muscle Regeneration: Leave It to the Macrophages!. Trends Immunol..

[31] Cianci R., Franza L., Massaro M.G., Borriello R., Tota A., Pallozzi M., De Vito F., Gambassi G. (2022). The Crosstalk between Gut Microbiota, Intestinal Immunological Niche and Visceral Adipose Tissue as a New Model for the Pathogenesis of Metabolic and Inflammatory Diseases: The Paradigm of Type 2 Diabetes Mellitus. Curr. Med. Chem..

[32] Tobin J.M., Gavitt B.J., Nomellini V., Dobson G.P., Letson H.L., Shackelford S.A. (2020). Immunotherapeutic options for inflammation in trauma. J. Trauma Acute Care Surg..

[33] Zamboni M., Nori N., Brunelli A., Zoico E. (2021). How does adipose tissue contribute to inflammageing?. Exp. Gerontol..

[34] Granic A., Sayer A.A., Robinson S.M. (2019). Dietary Patterns, Skeletal Muscle Health, and Sarcopenia in Older Adults. Nutrients.

[35] Rodrigues F., Domingos C., Monteiro D., Morouço P. (2022). A Review on Aging, Sarcopenia, Falls, and Resistance Training in Community-Dwelling Older Adults. Int. J. Environ. Res. Public Health.

[36] Mazzoccoli G. (2016). Body composition: Where and when. Eur. J. Radiol..

[37] Akazawa N., Okawa N., Hino T., Tsuji R., Tamura K., Moriyama H. (2020). Higher malnutrition risk is related to increased intramuscular adipose tissue of the quadriceps in older inpatients: A cross-sectional study. Clin. Nutr..

[38] Gioffrè-Florio M., Murabito L.M., Visalli C., Pergolizzi F.P., Famà F. (2018). Trauma in elderly patients: A study of prevalence, comorbidities and gender differences. Il G. Di Chir..

[39] Magee F., Wilson A., Bailey M., Pilcher D., Gabbe B., Bellomo R. (2021). Comparison of Intensive Care and Trauma-specific Scoring Systems in Critically Ill Patients. Injury.

[40] Kehoe A., Rennie S., Smith J.E. (2015). Glasgow Coma Scale is unreliable for the prediction of severe head injury in elderly trauma patients. Emerg. Med. J. EMJ.

[41] Jochems D., Leenen L.P.H., Hietbrink F., Houwert R.M., van Wessem K.J.P. (2018). Increased reduction in exsanguination rates leaves brain injury as the only major cause of death in blunt trauma. Injury.

[42] Brohi K., Gruen R.L., Holcomb J.B. (2019). Why are bleeding trauma patients still dying?. Intensive Care Med..

[43] Marsden M., Carden R., Navaratne L., Smith I.M., Penn-Barwell J.G., Kraven L.M., Brohi K., Tai N.R.M., Bowley D.M. (2018). Outcomes following trauma laparotomy for hypotensive trauma patients: A UK military and civilian perspective. J. Trauma Acute Care Surg..

[44] Harvin J.A., Maxim T., Inaba K., Martinez-Aguilar M.A., King D.R., Choudhry A.J., Zielinski M.D., Akinyeye S., Todd S.R., Griffin R.L. (2017). Mortality after emergent trauma laparotomy: A multicenter, retrospective study. J. Trauma Acute Care Surg..

[45] Seyfer A.E., Seaber A.V., Dombrose F.A., Urbaniak J.R. (1981). Coagulation changes in elective surgery and trauma. Ann. Surg..

[46] Nguyen R.K., Rizor J.H., Damiani M.P., Powers A.J., Fagnani J.T., Monie D.L., Cooper S.S., Griffiths A.D., Hellenthal N.J. (2020). The Impact of Anticoagulation on Trauma Outcomes: An National Trauma Data Bank Study. Am. Surg..

[47] Jenkins P.C., Dixon B.E., Savage S.A., Carroll A.E., Newgard C.D., Tignanelli C.J., Hemmila M.R., Timsina L. (2021). Comparison of a trauma comorbidity index with other measures of comorbidities to estimate risk of trauma mortality. Acad. Emerg. Med. Off. J. Soc. Acad. Emerg. Med..

[48] Elkbuli A., Yaras R., Elghoroury A., Boneva D., Hai S., McKenney M. (2019). Comorbidities in Trauma Injury Severity Scoring System: Refining Current Trauma Scoring System. Am. Surg..

[49] Ong A.W., Muller A., Sigal A., Fernandez F. (2019). Anemia at Discharge in Elderly Trauma Patients Is Not Associated with Six-Month Mortality. Am. Surg..

[50] Gungor O., Ulu S., Hasbal N.B., Anker S.D., Kalantar-Zadeh K. (2021). Effects of hormonal changes on sarcopenia in chronic kidney disease: Where are we now and what can we do?. J. Cachexia Sarcopenia Muscle.

