# Supplementary material for: Skeletal Muscle Quality Evaluation for Prognostic Stratification in the Emergency Department of Patients ≥65 Years with Major Trauma
Source: J Clin Med. 2025 Oct 23;14(21):7504. doi: 10.3390/jcm14217504 (PMC12608685; doi:10.3390/jcm14217504)
Supplement: Supplementary file 1 [file jcm-14-07504-s001.zip › jcm-3906085-supplementary.pdf]

Journal of
Clinical Medicine

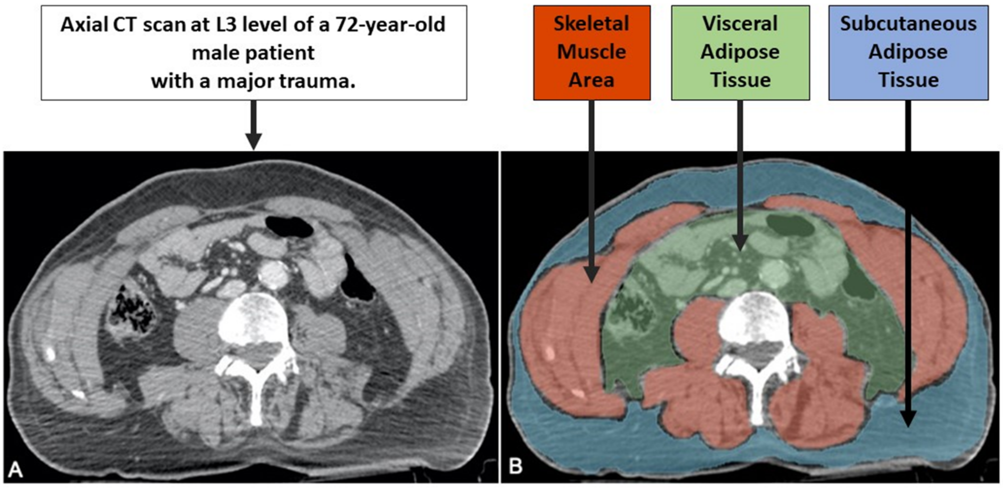

Supplementary Figure S1 – Abdominal CT scan at the level of 3rd lumbar in 72-year-old patients with major trauma sample (A). Figure (B) shows the distribution of skeletal muscle area, visceral adipose tissue, and subcutaneous adipose tissue.
